# Monkeypox Virus Evolution before 2022 Outbreak

**DOI:** 10.3201/eid2902.220962

**Published:** 2023-02

**Authors:** Eric Dumonteil, Claudia Herrera, Gilberto Sabino-Santos

**Affiliations:** Tulane University, New Orleans, Louisiana, USA (E. Dumonteil, C. Herrera, G. Sabino-Santos);; University of São Paulo, São Paulo, Brazil (G. Sabino-Santos)

**Keywords:** Monkeypox virus, MPXV, mpox, transmission, evolution, adaptation, viruses

## Abstract

Phylogenetic analysis of monkeypox virus genomes showed statistically significant divergence and nascent subclades during the 2022 mpox outbreak. Frequency of G>A/C>T transitions has increased in recent years, probably resulting from apolipoprotein B mRNA editing enzyme catalytic polypeptide 3G (APOBEC3) deaminase editing. This microevolutionary pattern most likely reflects community spread of the virus and adaptation to humans.

Monkeypox virus (MPXV) is a double-stranded DNA virus mostly associated with rodents and occasionally spilling over to humans, causing outbreaks of mpox (formerly monkeypox) that have been relatively short-lived and self-limiting because of ineffective transmission among humans (*1*). However, this view is challenged by reports that, since the start of the ongoing outbreak, in early April 2022, a total of 49,482 mpox cases in 94 countries had been confirmed (https://ourworldindata.org/monkeypox). Initial epidemiologic studies provided evidence of sustained human-to-human transmission in some non–MPXV-endemic countries in Europe, through close contacts, including in sexual networks (*2*). The first MPXV genome sequences from the outbreak were reported from Portugal on May 19, 2022 (*3*), and multiple additional sequences, which can shed light on virus circulation, are now available. Initial phylogenetic analyses indicated that the virus causing the 2022 outbreak belonged to MPXV clade II (formerly West African clade), which is less severe than clade I (formerly Congo Basin clade) (*4*), suggesting that the current outbreak was caused by the recent introduction of the virus into communities in non–MPXV-endemic countries (*2*). However, further analysis including additional MPXV genome sequences indicates a different scenario.

Indeed, phylogenetic analysis of 105 MPXV genomes (Appendix Table 1) revealed that viruses from 2022 belong to 2 clades that can be traced back to the previous 2017–2018 outbreak (Figure). One of those subclades, so far only identified in the United States (*5*), seems to have limited circulation (only 3 cases). All other 2022 viral genomes form a large monophyletic group, although a substantial level of sequence divergence among strains can already be detected, with several nascent subclades (Figure). Such divergence is not compatible with a recent diversification of the virus during the past few months of the outbreak. Rather, it reflects a continuous microevolution since the previous outbreak in 2017–2018. The most recent common ancestor for the 2022 outbreak can be traced back to around 20 years ago, at a rather similar time as the most recent common ancestor for the 2017–2018 outbreak. Furthermore, MPXVs from the 2022 outbreak are more closely related to strains that had been exported from Africa during the previous outbreak, rather than with strains circulating in Nigeria at that time. A strain from a person who traveled from Nigeria to Maryland, USA, in 2021 (*5*) can also be traced back to the root of the 2022 outbreak. Thus, the most likely scenario is that there has been silent and undetected circulation of MPXV, possibly including multiple non–MPXV-endemic countries outside Africa, since the 2017–2018 outbreak.

**Figure:**
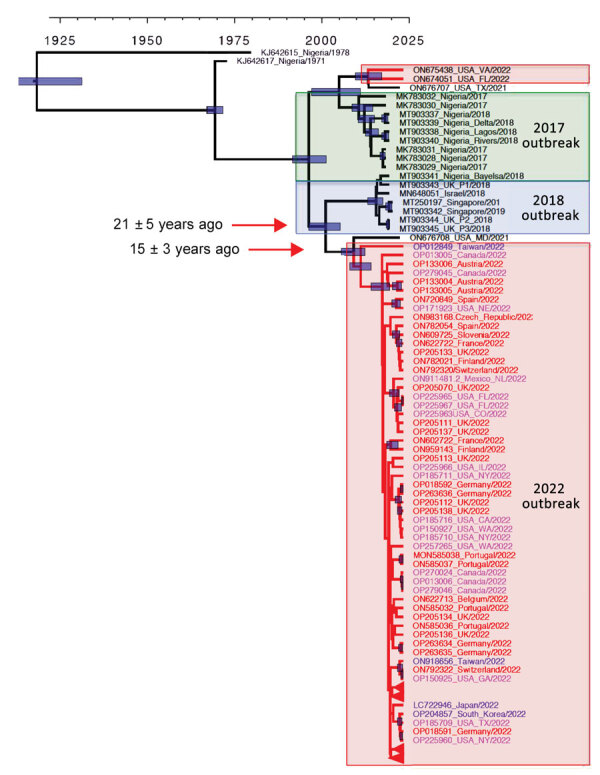
Origin of the 2022 oubreak of monkeypox virus (MPXV) infections. A) MPXV phylogeny of the 2022 outbreak, created by using 87 sequences from the 2022 outbreak together with sequences from past cases. Whole-genome Bayesian phylogeny and molecular estimates of divergence times were performed in BEAST 2.6.7 (https://beast.community) by using a generalized time reversible substitution model and a molecular clock of 5 × 10^–6^ substitutions/site/year under the assumption of constant population size. The model was run for 1 million generations and a burn-in of 10,000 trees. The maximum clade credibility tree is shown, and blue bars indicate uncertainty in node age for nodes with >50% support. Red indicates tree branches for 2022 strains. Sequence names for the 2022 outbreak are color-coded as follows: red, Europe; pink, North America; and blue, Asia.

Our observations raise the question of potentially increased adaptation of current virus strains to humans. Variations in genomic content may shape the evolution of orthopoxviruses, and gene gain/loss may correlate with pathogenicity and host adaptation (*6*). We found multiple genomic changes in the MPXVs from 2022; at least 51 single-nucleotide polymorphisms (SNPs) differentiated the first 18 viral genomes from the 2022 outbreak from those from 2017–2018 (Appendix Table 2) and a few larger insertions/deletions. Of the 51 SNPs, 26 caused amino acid changes and 21 were synonymous substitutions. Additional SNPs can be detected among genome sequences from 2022, underlying the established divergence within the outbreak (Appendix Table 2). Those changes may be associated with mutational pressure and adaptation (*7*,*8*), and future studies should help assess their phenotypic effects.

Further analysis of the substitutions showed major bias in their distribution in viruses from 2022; 61/70 (87.1%) of all substitutions were G>A/C>T transitions, followed by 6/70 (8.6%) T>C/A>G transitions, and 2/70 (2.8%) were C>A/G>T transversions and 1/70 (1.4%) A>C/T>G transversions. Comparison of these substitution proportions with those observed in clade II up to 2018 and those from the clade I showed a striking pattern (Appendix Figure). Indeed, viruses from both clades had nearly identical proportions of substitution types before 2018, and the proportion of G>A/C>T transitions in clade II viruses from 2022 had doubled (χ^2^ = 55.3; p<0.0001) (Appendix Figure). Those considerable changes in substitutions most likely reflect the editing activity of the human APOBEC3G enzyme (apolipoprotein B mRNA editing enzyme, catalytic subunit 3G**)**, which catalyzes strand-specific C>U deamination, resulting in G>A substitutions in the complementary strand of viral genomes (A. O’Toole, unpub. data, https://virological.org/t/initial-observations-about-putative-apobec3-deaminase-editing-driving-short-term-evolution-of-mpxv-since-2017/830; [*9*]).

In conclusion, our analyses of MPXV genome sequences indicate that the virus has been circulating silently and undetected for about 2 decades, probably in multiple non–MPXV-endemic countries outside of Africa. Also, a clear genomic signature of a recent change in hosts is evidenced by major changes in its nucleotide substitution pattern. Our observations have major public health implications; the changing epidemiology of MPXV infections and human circulation of the virus in non–MPXV-endemic countries call for increased surveillance (*1*). The public health crisis caused by the COVID-19 pandemic may have favored the spread of MPXV under the radar in the past few years; however, the existence of asymptomatic carriers cannot be ruled out and may have contributed to the undetected spread of MPXV.

Appendix Additional information for study of monkeypox virus evolution before the 2022 outbreak.
