# Supplementary material for: Monkeypox Virus Evolution before 2022 Outbreak
Source: Emerg Infect Dis. 2023 Feb;29(2):451–3. doi: 10.3201/eid2902.220962 (PMC9881786; doi:10.3201/eid2902.220962)
Supplement: Appendix — Additional information for study of monkeypox virus evolution before the 2022 outbreak. [file 22-0962-Techapp-s1.pdf]

Article DOI: https://doi.org/10.3201/eid2902.220962

Monkeypox Virus Evolution before 2022 Outbreak

Appendix

Appendix Table 1. List of MPXV genome sequences used

Accession #	Country	Year
AF380138	Zaire	
AY603973	WRAIR7-61	
AY741551	Sierra Leon	2005
AY753185	Denmark	1958
DQ011153	USA	2003
DQ011154	Congo	2002
DQ011155	Zaire	1979
DQ011156	Liberia	1970
DQ011157	USA	2003
HM172544	Zaire	1979
HQ857562	Zaire	
HQ857563	D14L knockout	2007
ITM_MPX_1	Belgium	2022
JX878407	DRC	2006
JX878408	DRC	2006
JX878409	DRC	2006
JX878410	DRC	2006
JX878411	DRC	2006
JX878412	DRC	2006
JX878413	DRC	2006
JX878414	DRC	2006
JX878415	DRC	2006
JX878416	DRC	2006
JX878417	DRC	2006
JX878418	DRC	2007
JX878419	DRC	2007
JX878420	DRC	2007
JX878421	DRC	2007
JX878422	DRC	2007
JX878423	DRC	2007
JX878424	DRC	2007
JX878425	DRC	2007
JX878426	DRC	2007
JX878427	DRC	2007
JX878428	DRC	2007
JX878429	DRC	2007
KC257459	Sudan	2005
KC257460	DRC	1985
KJ642612	Zaire	1986
KJ642613	Zaire	1978
KJ642614	Netherlands	1965
KJ642615	Nigeria	1978
KJ642616	France	1968
KJ642617	Nigeria	1971
KJ642618	Cameroon	1990
KJ642619	Gabon	1988
KP849469	DRC	2008
KP849470	Côte d’Ivoire	1971
KP849471	DRC	1985
LC722946	Japan	2022
MK783028	Nigeria	2017
MK783029	Nigeria	2017
MK783030	Nigeria	2017

Accession #	Country	Year
MK783031	Nigeria	2017
MK783032	Nigeria	2017
MN346690	Côte d’Ivoire	2017
MN346691	Côte d’Ivoire	2017
MN346692	Côte d’Ivoire	2017
MN346693	Côte d’Ivoire	2017
MN346694	Côte d’Ivoire	2017
MN346695	Côte d’Ivoire	2017
MN346696	Côte d’Ivoire	2017
MN346697	Côte d’Ivoire	2017
MN346698	Côte d’Ivoire	2017
MN346699	Côte d’Ivoire	2017
MN346700	Côte d’Ivoire	2017
MN346702	Côte d’Ivoire	2018
MN648051	Israel	2018
MN702448	CAR	2018
MT250197	Singapore	2019
MT903337	Nigeria	2018
MT903338	Nigeria	2018
MT903339	Nigeria	2018
MT903340	Nigeria	2018
MT903341	Nigeria	2018
MT903342	Singapore	2019
MT903343	UK	2018
MT903344	UK	2018
MT903345	UK	2018
MT903346	USA	2003
MT903347	USA	2003
MT903348	USA	2003
NC003310	Zaire	1996
ON563414	USA	2022
ON568298	Germany	2022
ON585029	Portugal	2022
ON585030	Portugal	2022
ON585031	Portugal	2022
ON585032	Portugal	2022
ON585033	Portugal	2022
ON585034	Portugal	2022
ON585035	Portugal	2022
ON585036	Portugal	2022
ON585037	Portugal	2022
ON585038	Portugal	2022
ON595760	Switzerland	2022
ON602722	France	2022
ON609725	Slovenia	2022
ON615424	The Netherlands	2022
ON622712	Belgium	2022
ON622713	Belgium	2022
ON649879	Israel	2022
ON674051	USA	2022
ON675438	USA	2022
ON676707	USA	2021
ON676708	USA	2021
ON720849	Spain	2022
ON782021	Finland	2022
ON782054	Spain	2022
ON792320	Switzerland	2022
ON792322	Switzerland	2022
ON843166	Portugal	2022
ON843168	Portugal	2022
ON911481	Mexico	2022
ON918656	Taiwan	2022
ON959143	Finland	2022
ON983168	Czech Republic	2022
OP012849	Taiwan	2022
OP013005	Canada	2022
OP013006	Canada	2022
OP018591	Germany	2022
OP018592	Germany	2022

Accession #	Country	Year
OP133004	Austria	2022
OP133005	Austria	2022
OP133006	Austria	2022
OP150925	USA	2022
OP150927	USA	2022
OP160532	The Netherlands	2022
OP171922	USA	2022
OP171923	USA	2022
OP185709	USA	2022
OP185710	USA	2022
OP185711	USA	2022
OP185715	USA	2022
OP185716	USA	2022
OP185717	USA	2022
OP204857	South Korea	2022
OP205069	UK	2022
OP205070	UK	2022
OP205111	UK	2022
OP205112	UK	2022
OP205113	UK	2022
OP205133	UK	2022
OP205134	UK	2022
OP205135	UK	2022
OP205136	UK	2022
OP205137	UK	2022
OP205138	UK	2022
OP205139	UK	2022
OP225960	USA	2022
OP225963	USA	2022
OP225965	USA	2022
OP225966	USA	2022
OP225967	USA	2022
OP225968	USA	2022
OP225969	USA	2022
OP257252	USA	2022
OP257253	USA	2022
OP257264	USA	2022
OP257265	USA	2022
OP263634	Germany	2022
OP263635	Germany	2022
OP263636	Germany	2022
OP270024	Canada	2022
OP279045	Canada	2022
OP279046	Canada	2022
OP289782	Peru	2022
OP289783	Peru	2022

*We thank the respective authors for making these sequences publicly available. This work does not involve human or animal subjects and is exempt from IRB/IACUC review.

Appendix Table 2. SNP signatures differentiating MPXV from 2022 from the previous outbreak*

Affected sequences	SNP†	Gene/Protein	AA change‡
all 2022	G1263A	gp001	S105L
all 2022	G2597A	gp002	S54F
all 2022	G3117A	gp003	D264N
all 2022	G3528A	gp003	no change
all 2022	C3824T	gp003	no change
all 2022	C8281T	gp006	no change
all 2022	C14512T	gp012	A423D
all 2022	C15941A	Intergenic	-
all 2022	G22246A	gp025	no change
all 2022	G26184A	gp029	S36F
all 2022	G30890A	gp035	R48C
all 2022	G31576A	gp035	no change
all 2022	G34982A	gp041	P78S
all 2022	G37725A	gp044	E125K
all 2022	G38883A	gp044	no change
all 2022	C39185T	gp044	no change
all 2022	C39642T	gp045	no change
all 2022	C39662T	gp045	E353K
all 2022	T40290C	gp045	no change
all 2022	T44450C	gp051	K658E
all 2022	G54642A	gp057	L108F
all 2022	G64824A	gp068	no change
all 2022	C73593T	gp078	S30L
all 2022	G73766A	gp078	D88N
all 2022	G74732A	gp079	M142I
all 2022	G77911A	gp083	E162K
all 2022	G81803A	gp090	no change
all 2022	C82901T	gp090	no change
all 2022	G82979A	gp090	no change
all 2022	C85115T	gp090	no change
all 2022	G95562A	gp100	no change
all 2022	G124660A	gp129	E62K
all 2022	G125204A	gp129	R243Q
all 2022	C129228T	gp134	S307L
all 2022	C151028T	gp157	H221Y
all 2022	A152029C	Intergenic	-
all 2022	A152497G	gp159	K141E
all 2022	G156363A	Intergenic	-
all 2022	G162811A	gp165	no change
all 2022	C162899T	gp165	no change
all 2022	G170835A	gp172	no change
all 2022	T171146C	gp172	L179S
all 2022	G178847A	Intergenic	-
all 2022	G183108A	gp182	D210N
all 2022	C184647T	gp182	no change
all 2022	G187706A	gp182	M1741I
all 2022	G194770A	gp189	D266N
all 2022	c195066T	gp189	no change
all 2022	c195477T	gp189	no change
all 2022	c195997T	gp190	S54F
all 2022	c197326T	gp191	S105L
Unique SNPs within 2022 MPXV genomes			
UZ REGA1 Belgium	insert A 171	Intergenic	-
ON585038, Portugal	Insert TT 595-596	Intergenic	-
ON595760, Switzerland	Insert T 617	Intergenic	-
ON595760, Switzerland	multiple changes	gp002	H79P, S85I, Q94R, E96K, frameshift
ON595760, Switzerland	Insert A 4646	Intergenic	-
PT008	C17490T	gp014	no change
ON595760, Switzerland	G19465A	gp021	S66L
ON602722, France	Del A 44024	Intergenic	-
UZ REGA1 Belgium	C46648T	gp952	R120K
ON595760, Switzerland	insert A 51344	gp056	frameshift
ON585033, Portugal	C55600T	gp059	D24N
ON609725, Slovenia	g55658a	gp059/gp060	no change
ON609725, Slovenia	del A55650	gp059	frameshift
ON602722, France	del A55650	gp059	frameshift
ON595760, Switzerland	insert A 58277	gp062	frameshift
ON609725, Slovenia	C64953T	gp068	no change

Affected sequences	SNP†	Gene/Protein	AA change‡
ON563414, USA	insert A 77781	gp083	frameshift
ON585037, Portugal	G83381A	gp090	no change
ON602722, France	C90434T	gp095	S92F
ON602722, France	del T 95317	gp100	framshift
ON602722, France	G95326A	gp100	E47K
ON595760, Switzerland	insert C 102112	gp105	frameshift
ON595760, Switzerland	insert T 122925	gp127	frameshift
ON585032, Portugal	insert ATC 137049	gp138	insert D372
ON602722, France	G151117T	Intergenic	-
NL001_2022, Netherlands	G156179A	Intergenic	-
ON609725, Slovenia	G168089A	gp170	E62K
NL001_2022, Netherlands	G171578A	gp173	E6K
ON602722, France	insert A 174618	Intergenic	-
ON585037, ON585038, Portugal,	G177537A	gp178	E390K
ON585037, ON585038, Portugal,	G182495A	gp182	no change
ON568298, Germany	C188282T	Intergenic	
ON609725, Slovenia	G191789A	gp187	R84K
ON595760, Switzerland	insert A 197489	gp191	frameshift

*Analysis based on the first 18 MPXV genome sequences released.
†Position of SNPs based on Singapore 2019 genome sequence (accession #MT903342).
‡AA: amino-acid, position given within each viral protein.

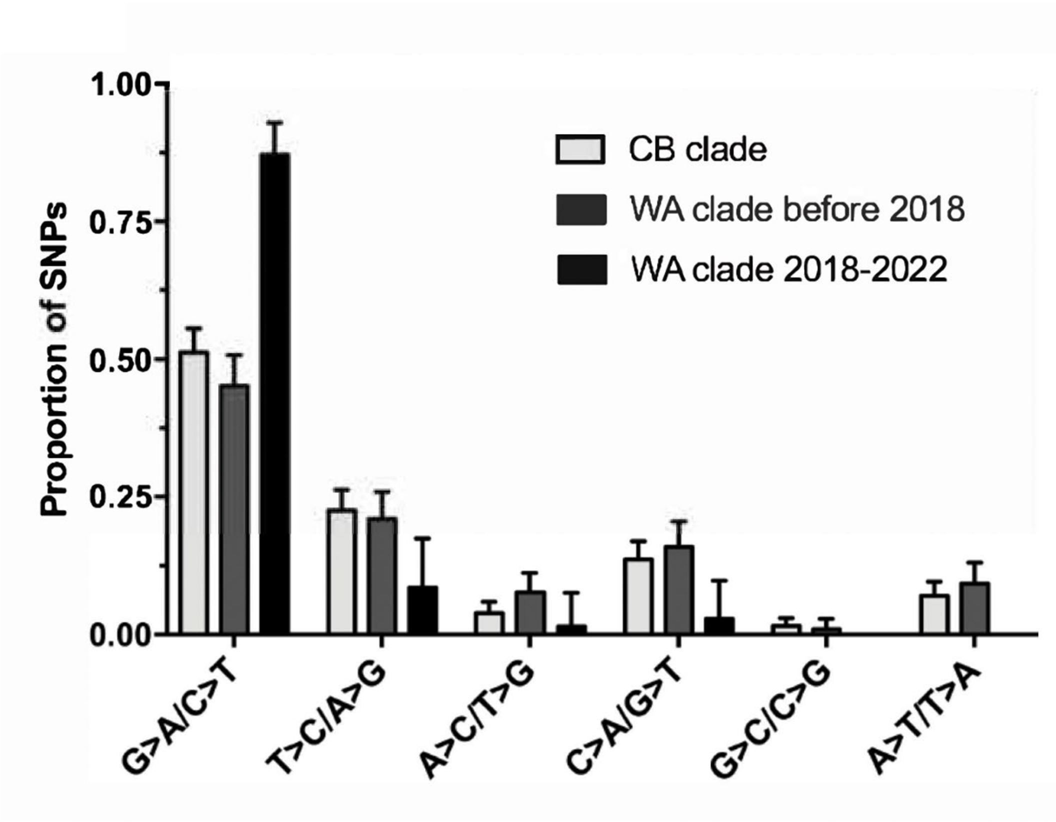

Appendix Figure. Distribution of substitution proportions. The proportion of substitutions (± 95% CI) according to their type was recorded among MPXV genomes from the 2022 outbreak (clade II 2018–2022, 18 sequences), and compared with the substitution pattern in clade II prior to 2018 (clade II before 2018, 23 sequences), and to clade I (40 sequences). A significant change in substitution pattern can be detected in recent years (χ^2=55.3, P<0.0001), and the proportion of G>A/C>T transitions almost doubled in viruses from the 2022 outbreak.
